# Clarithromycin Dissolution Enhancement by Preparation of Aqueous Nanosuspensions Using Sonoprecipitation Technique

**Published:** 2014

**Authors:** Es ׳hagh Esfandi, Vahid Ramezani, Alireza Vatanara, Abdolhossein Rouholamini Najafabadi, Seyyed Pouya Hadipour Moghaddam

**Affiliations:** a Department of Pharmaceutics, Faculty of Pharmacy, Tehran University of Medical Sciences, Tehran, Iran.; b Department of Pharmaceutics, Faculty of Pharmacy, Shahid Sadoughi University of Medical Sciences, Yazd, Iran.

**Keywords:** Clarithromycin, Nanosuspension, Solubility, Stabilizer, Antimicrobial activity

## Abstract

Clarithromycin (CLM) is a member of macrolide family with broad spectrum antibiotic activity. It is practically insoluble in water and its poor solubility is pH dependent. In this study, series of nanosuspensions containing CLM and stabilizer such as HPMC, NaCMC, polysorbate 80, poloxamer 188 and polyvinyl alcohol in various ratios were prepared using sonoprecipitation method. Briefly, CLM was dissolved in acid solution and the pH of solution was raised under sonication and the effects of different stabilizers on particle size of nanoparticles were evaluated. Characterization of nanoparticles in terms of size, polydispersity index, zeta potential, differential scanning calorimetery and dissolution studies was performed. Antimicrobial activity of CLM nanosuspension was compared with coarse powder by using an agar well diffusion method. The results showed that HPMC was more efficient in size reduction of particles and presence of HPMC E_5_ with ratio of 3:5 to CLM in formulation led to develop the stable nanosuspension with particle size of 340 nm. The obtained nanosuspension successfully showed enhanced dissolution rate and antimicrobial activity.

## Introduction

One of the major challenges in pharmaceutical industries is the poor water solubility of many drugs, which causes delivery limitations(1-2). The size and size distribution of pharmaceutical particles significantly affect the dissolution rate and therefore the bioavailability. Generally, fine particles with higher specific surface areas dissolve rapidly and act more efficiently. For this reason, formation of micro and nanoparticles and controlling the size distribution of pharmaceutical particles is an important strategy (3). In the recent years, nanosuspensions have been introduced to the pharmaceutical markets as novel dosage forms with improved physicochemical and biological properties. Nanosuspension is a liquid dispersion containing drug nanoparticles that is stabilized by some suitable polymers and/or surfactants (4-5). As described by Noyes–Whitney equation, the particle size reduction can lead to an increased dissolution rate due to greater surface area. Also, according to Kelvin and Ostwald-Freundlich equation, the saturation solubility could be increased due to the amplified dissolution pressure. Moreover, increased adhesiveness to surfaces/cell membranes is other special characteristic of nanosuspensions (6-8). 

Based on technical aspect, another outstanding feature of nanosuspensions is diversity of methods used for preparation. Generally, the existing processes for producing nanosuspension can be classified into top-down and bottom-up processes.

The top-down process involves comminution technologies such as air jet or wet milling and high pressure homogenization for reducing the particle size (9-10). Although, these methods have been used for some commercial products, but none of them are efficient because of possible pharmaceutical contaminations and degradations during grinding processes (11).

Bottom–up methods are based on precipitation technologies such as supercritical fluid processing, spray freezing/evaporation into liquid and the liquid solvent quenching processes (12-17).

Nanosuspensions are mostly stabilized by means of appropriate steric and electrostatic stabilizers in proper quantities. Commonly used steric stabilizer includes hydroxypropyl methyl cellulose (HPMC), hydroxypropyl cellulose (HPC), polyvinylpyrrolidone (PVP K-30), and poloxamer (188 and 407), while electrostatic stabilizers includes polysorbates (mainly Tween 80) and sodium lauryl sulphate (SLS) (18).

**Figure 1:**
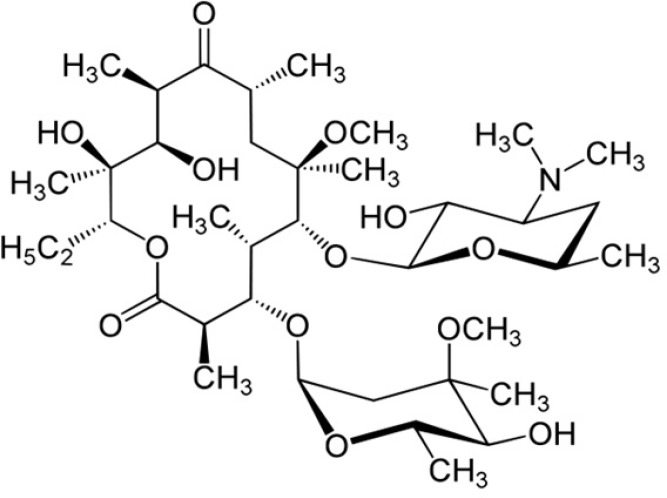
molecular structure of CLM

Clarithromycin (CLM) is a semi-synthetic macrolide antibiotic with a 14-member ring (Figure 1) and is a broad spectrum antibiotic that seems to be effective against Gram positive and respiratory tract infectious bacteria as like as *Pseudomonas aeruginosa*, *Klebsiella pneumoniae, Mycoplasma pneumonia*, *Streptococcus pneumonia*, *Haemophilus influenza *(19-21).

According to the biopharmaceutical classification system (BCS), CLM is considered a class II molecule, with low solubility and high permeability *in-vivo*. The water solubility of drug is about 0.342 μg/mL, but it increases in acidic media owing to ionization of dimethylamino moiety (Figure 1) as the only ionizable group (pKa 8.8) (22).

Several studies such as application of cyclodextrins and formation of emulsions, liposomes and nanoparticles have been performed to improve the solubility and other physicochemical properties of CLM (23-26).

The aim of present study was to evaluate a feasible method employing some commercial excipients for production of non-agglomerated CLM nanosuspensions for enhancement in its physicochemical and biological properties.

## Experimental

*Materials*

Clarithromycin USP was provided by Shafa Pharmed (Iran). HPMC (Methocel) E_5_, E_15_ and E_50_ were obtained from Dow Chemical (USA) and pluronic F_68_ (poloxamer 188) was purchased from BASF (USA). Poly vinyl alcohol (PVA) (MW 30-70 kDa) and Sodium carboxymethyl cellulose (NaCMC) were obtained from Sigma-Aldrich (USA). Tween 80 was purchased from Fisher Chemical Company (USA). Ortho-phosphoric acid (85%) and all other chemicals of analytical grade were supplied by Merck (Germany).

*Preparation of nanosuspensions*

Formulations of CLM nanosuspension were prepared using 50 mg CLM and various ratios of stabilizers according to Table 1. At first, CLM was dissolved in aqueous phosphoric acid solution (final pH 4.0). The pH of solution was raised by NaOH (10 M) under sonication in an ice bath. A Hielscher sonicator (UP400S) with tip diameter of 3 mm, and length of 100 mm was applied to produce sonication waves. The precipitation was performed in 100% amplitude. In this conditions the medium was faced to fixed waves with frequency of 24 kHz and the power of 400 W. Addition of NaOH was continued until the solution became completely cloudy. The nanosuspensions were precipitated by centrifugation in 6000 rpm and washed by deionized water in order to remove the residual stabilizer. The retrieved nanoparticles were frozen at -20 °C and finally were lyophilized (Christ, Germany) at -40 °C for 48 h.

**Table 1:** Various formulation of nanpsuspension containing 50 mg CAM with different concentration of stabilizers.

**Stabilizer**	**Stabilizer:CLM **					
	**1:10**	**1:5**	**2:5**	**3:5**	**4:5**	**1:1**
**NaCMC**		F_1_				F_2_
**Poloxamer 188**		F_3_				F_4_
**Tween 80**		F_5_				F_6_
**PVA**		F_7_				F_8_
**HPMC E**_5_	F_9_	F_10_	F_11_	F_12_	F_13_	F_14_
**HPMC E**_15_	F_15_	F_16_	F_17_	F_18_	F_19_	F_20_
**HPMC E**_50_	F_21_	F_22_	F_23_	F_24_	F_25_	F_26_

*Particle size measurement*

Mean particle size (Z average) and particle size distribution in the case of polydispersity index (PDI) were performed by photon correlation spectroscopy (Zetasizer^®^, Malvern Instruments, UK). Particle sizes were measured in aqueous medium at room temperature without any further dilution. In addition to particle size, this instrument was used to measure the zeta potential of aqueous suspensions by determining the electrophoretic mobility of particles. All measurement was taken in triplicates.

*Scanning electron microscopy (SEM)*

Selected lyophilized nanoparticles were mounted onto an aluminum stub and sputter-coated for 90 s with gold DS-Sputtering (HUMMER-II, TECHNICS, USA). The morphology of CLM nanoparticles was examined by a scanning electron microscope (S-4160, HITACHI, JAPAN) at an acceleration voltage of 15 kV.

*Physical stability studies*

Physical stability of CLM nanosuspensions was studied at 25 °C for 14 days (2 weeks) according to previous studies (27-28). Three batches of each formulation were applied for analyzing the CLM nanosuspensions particle size and polydispersity index (PDI) as the key parameters in evaluation of physical stability (29).

*Dissolution studies*

Dissolution experiments were carried out on commercial suspension of CLM and lyophilized nanoparticles. Briefly, 50 mg of powder was poured in 50 mL phosphate buffer (pH 7.0) and stirred on a magnetic stirrer at 50 rpm at room temperature. Samples were withdrawn at defined intervals and filtered through 0.22 μm filters. The amount of dissolved CLM was determined using Waters series HPLC (Waters, USA). The column was Teknokroma, lichrospher C_18_ (150 mm × 4.6 mm) and the mobile phase consisted of methanol and 0.067 M monobasic potassium phosphate with ratio of 13 to 7 and pH was adjusted to 4.0 by using phosphoric acid. Sample injection volume was 20 μL and detecting wavelength was 205 nm. The analysis was performed in triplicates.

*Thermal analysis*

A differential scanning calorimeter (Mettler-Toledo, Switzerland) was used to thermal analysis of CLM nanostructures, coarse powder and other additives. About 5-10 mg of lyophilized nanoparticles or other samples were sealed in aluminum pans and were heated from 10 to 300 °C at the rate of 10 °C/min.

*Antimicrobial activity*

The antimicrobial activity of CLM nanosuspension was compared with coarse suspension of CLM (Sandoz^®^) by using an agar well diffusion method. Different target bacteria were inoculated and cultivated on top of caso agar and circular wells were cut in the agar culture media. 0.1 mL of suspended samples were added in equal concentrations to wells. The zone of inhibition (ZOI) was measured after 24 h of incubation at 37 °C.

**Figure 2:**
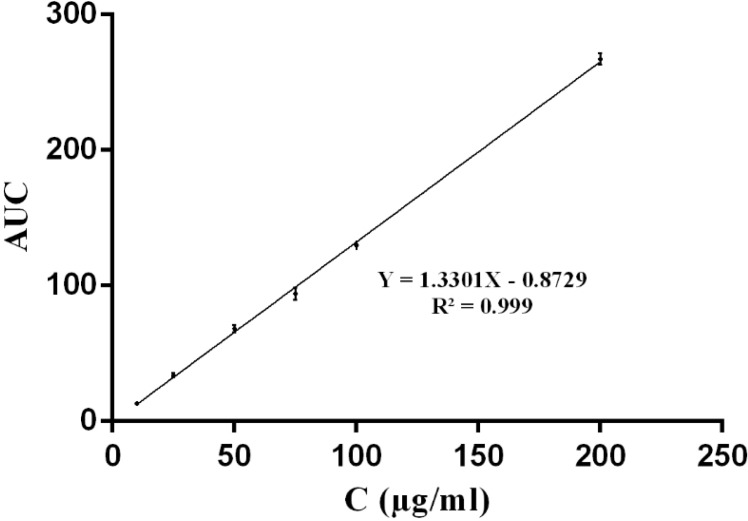
Calibration curve for determination of of CLM using HPLC method

## Results and Discussion

CLM nanoparticles were prepared using ultrasound nanoprecipitation technique. In this process has been successfully used for production of different nanostructures, ultrasound irradiation induces homogeneous agitation in aqueous media (30-31). The acoustic cavitation in the liquid which induced by ultrasound, mainly results in diffusion of energy in liquid media. In this way, ultrasound leads to bubble collapse in the liquid and lead to concentration of high amounts of local energy.

Production of nanoparticles was performed by fixing the method variables and aimed to screen various stabilizer (as a critical variable) to obtain most favorable particle size and size distribution index (PDI). In this way, a range of stabilizers including ionic surfactant, non ionic surfactant and semi synthetic polymers in various ratios were examined.

**Figure 3:**
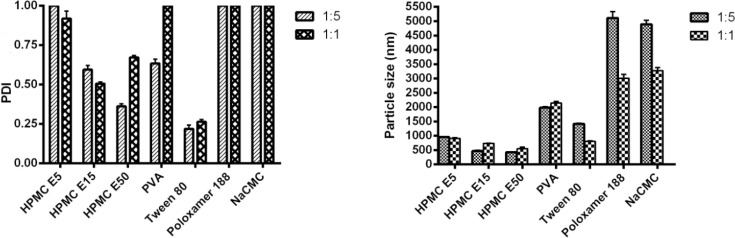
Effect of different stabilizers in ratios of 1:5 and 1:1 on CLM particle sizes and PDI

Figure 3 demonstrates the effects of different ingredients in 1:5 and 1:1 ratios of stabilizer to drug. As shown in this figure, application of 1:1 ratios of Tween 80 and poloxamer 188 as non-ionic surfactants resulted in nanosuspension with particle size of 805.60 ± 22.5 nm and 3011.0 ± 133.5 nm respectively. In the same way, using of 1:1 NaCMC as an ionic surfactant and PVA as a polymeric surfactant resulted in the formation of suspensions containing agglomerated large particles (3277.00 ± 102.4 nm for NaCMC and 2144.00 ± 58.7 nm for PVA). On the other hand, all of applied HPMC types showed a better effect on particle size reduction during sonopercipitation and produced particles in size range from 956.00 to 424.15 nm.

Regarding that CLM is a macrolide with lots of hydroxyl groups that potentially can generate lots of hydrogen bonds with other molecules, coverage of nuclei with stabilizer would have inhibitory effects for other molecules to reach to nuclei and inhibit the particle growth (32).

Structure of HPMC contains too many hydroxyl groups which can form lots of hydrogen bonds with CLM molecules on the nucleus surface. In contrast, there is just one hydroxyl group per one monomer in PVA structure and also, there is just one ether group in one monomer of Poloxamer 188.

Similarly, insufficient effect of NaCMC on size reduction might be attributed to the excessive repulsive force between negative charge of the polymer and CLM particles (with zeta potential equal to -7). In addition, negative charge of both CLM nanostructures and NaCMC increased simultaneously with increasing pH.

*Effect of different grades of HPMC on*

*particle size and stability*

**Figure 4:**
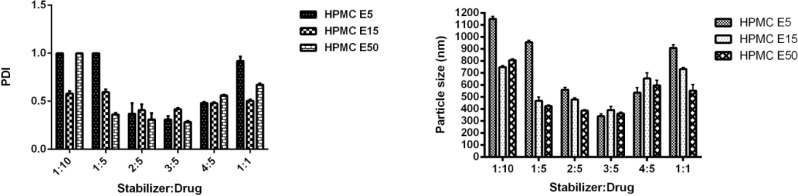
Effect of different HPMC grades and ratios on CLM particle size and PDI

As presented in Figure 4, application of HPMC in three grades and various concentrations provided similar patterns in particle size and PDI of CLM nanosuspensions. In order to evaluate the effect of stabilizer ratio on CLM particle size, ratios of stabilizer to CLM from 1:10 to 1:1 were investigated. Different ratios of all HPMC grades drastically influenced particle size as well as polydispersity index (PDI). Incrementing the ratio of HPMC E_5_ up to 3:5 led to reduction in particle size to a minimum (340.0 ± 20.6 nm). HPMC E_15_ as well as HPMC E_50_ demonstrated similar behavior with slightly larger particle size(E_15_ = 391.4 ± 31.0 and E_50_ = 362.7 ± 11.8 nm). Surprisingly, increasing the ratio of stabilizer up to 1:1 caused to an increase in particle size to 908.6 ± 27.0 for HPMC E_5_, 733.7 ± 11.6 for HPMC E_15_ and 553.5 ± 51.0 for HPMC E_50_. Increasing in particle size with incrementing the HPMCs ratio may be attributed to the concurrent increase in solution viscosity, where, the ultrasound mediated micro mixing and liquid vortex is more difficult in viscose media (33).

*Physical stability studies*

Nanosuspensions containing HPMCs as stabilizers were stored at 25 °C for 2 week and samplings were performed in the times of zero, 1, 3 and 14 days. Stability of nanoparticles was investigated by measuring the mean particle size and PDI. 

**Figure 5:**
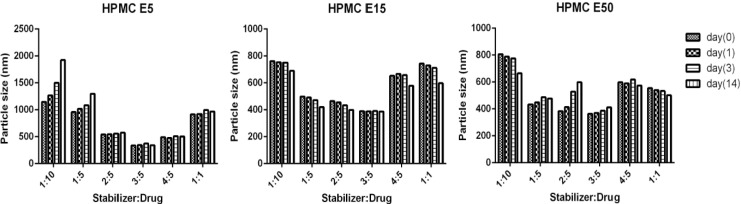
Effect of different stabilizers on particle size of CLM during stability studies

**Figure 6:**
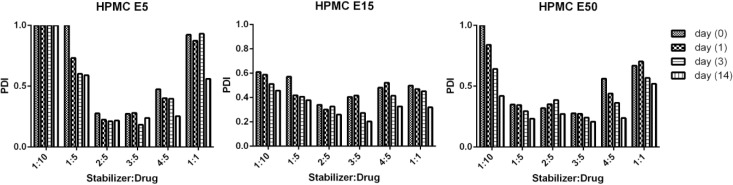
Effect of different stabilizers on PDI of CLM nanoparticles during stability studies

As shown in Figures 5 and 6, presence of HPMCs in nanosuspensions efficiently preserved CLM particles from aggregation and agglomeration during incubation. Although, high increase in particle size was seen in formulations F_9_ and F_10_ containing lower ratios of HPMC E_5_, but increase in concentration of polymer significantly inhibited aggregation. The inhibitory effects of stabilizer on particle agglomeration in high concentrations may be due to formation of permanent steric barrier around particles. 

On the contrary, a moderate decrease in particle size of formulations F_19_, F_20_, F_25_ and F_26_ during 14 days was observed. This particle size reduction during incubation maybe related to trivial solubilization of CLM from surface of nanoparticles in high concentrations of stabilizer and creation of new nucleus.

*Dissolution studies*

**Figure 7:**
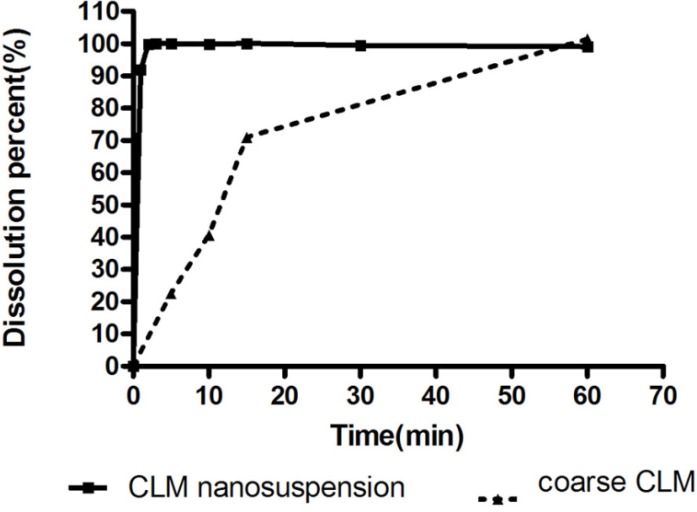
Dissolution profile of CLM coarse suspension and nanosuspension

Dissolution enhancement is a common rationale in preparation of nanostructures of various poorly soluble drugs. In order to evaluate the effect of size on dissolution profile of CLM, studied dissolution rate of nano and commercial suspensions of CLM were compared. As shown in Figure 7, almost 100% of nanoparticles were dissolved temporarily. In contrast, it took 15 minutes for dissolution of about 70% of coarse particles and 100% of drug was dissolved during 1 hour.

Higher surface area of nanoparticles in comparison with coarse particles results in enhancement of dissolution rate. According to Noyes-Whitney dissolution rate law (34) increasing the particle surface area is an effective way to increase dissolution rate. Superior dissolution of CLM nanostructures can potentially improve bioavailability and other drug performances.

*Morphological and thermal analysis*

**Figure 8:**
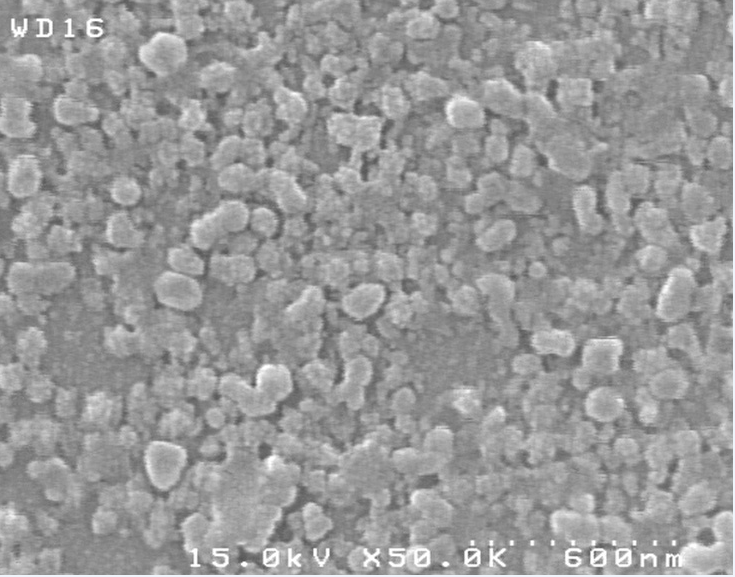
SEM micrograph of CLM nanoparticles

**Figure 9:**
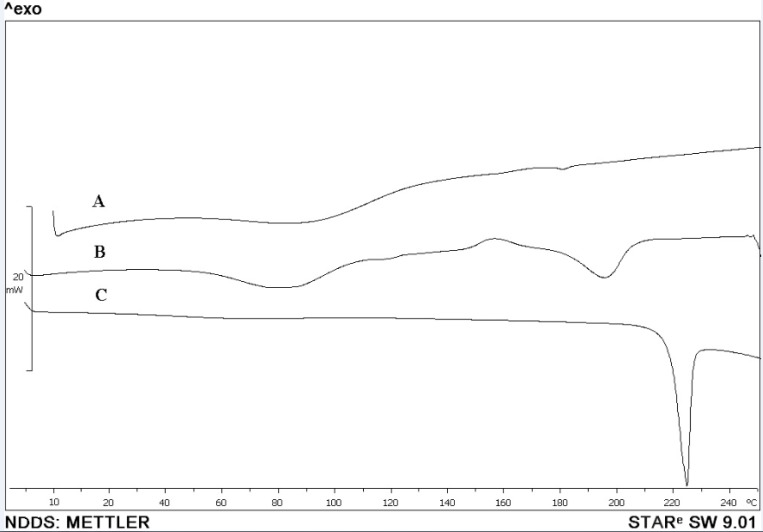
DSC thermogram of coarse and nanosuspension forms of CLM. A; thermogram of HPMC E_5_, B; lyophilized nanosuspension (F_12_) and C; coarse CLM powder

SEM micrograph of dried nanosuspension (Figure 8) illustrates irregular shaped particles in nano scale sizes. In Figure 9, the endotherm at 225 °C for unprocessed CLM indicated melting point. On the other hand, DSC thermogram of pure HPMC didn’t show any peak from 10 to 300 °C (Figure 9). Thermogram of washed and freeze dried nanoparticles showed a broad peak with onset temperature of 180 °C. Change in endothermic pick of nanosuspension can be related to the formation of amorphous or another polymorph of CLM during formation of nanostructures or presence of trivial residues of HPMC around

the nanoparticles. In general, formation of amorphous nanoparticles could be effective in the increase of dissolution rate (35). Further investigation is required to exact determination of the difference between dissolution rate of amorphous and crystal form of CLM nano particles.

*Antimicrobial activity*

The activity of nano and coarse suspensions on *Staphylococcus aureus, Pseudomonas* *aeruginosa *and *Klebsiella pneumonia *as three susceptible bacteria were evaluated by means of well diffusion method. Obtained results are summarized in Table 2 which shows that similar behavior were repeated for Gram negative bacteria like *P. aeruginosa *and *K. pneumonia *and Gram positive one such as *S. aureus*. All together, the antimicrobial effects of nanosuspension were significantly higher than coarse CLM. Higher antibacterial activity of CLM nanoparticles could be attributed to its higher dissolution rate, and subsequently increase in CLM diffusion in culture media during exponential growth of bacteria.

**Table 2:** Inhibitory zone (mm) of formulations on microbial culture

**Organism**	**Mean diameter of growth inhibition (mm)** **± SD**		
	**CLM nanosuspension( F**_12_ **)**	**CLM coarse powder**	**Deionized water**
***Staphylococcus aureus*** **ATCC 25923**	27.67± 0.58	20.33± 0.58	0.00± 0.00
***Pseudomonas aeruginos*** **ATCC 27853**	22.33± 1.53	16.00± 1.00	0.00± 0.00
***Klebsiella pneumoniae*** **ATCC 13883**	20.33± 0.58	15.00± 1.00	0.00± 0.00

## Conclusion

The results of this study showed that formation of CLM nanoparticles enhanced physicochemical and antibacterial properties of this drug. It was demonstrated that the type of stabilizers as well as their ratio can efficiently influence characteristics of CLM particles in nanosuspension. Application of various grades of HPMCs in sonoprecipitation of CLM significantly decreased particle size and stabilized the formulations at least for 14 days at room temperature. The resulted nanoparticles could be proposed for further processing to be employed by different drug delivery routes.
